# Examining bi-directional links between loneliness, social connectedness and sleep from a trait and state perspective

**DOI:** 10.1038/s41598-024-68045-y

**Published:** 2024-07-27

**Authors:** Christine Dworschak, Thomas Mäder, Charlotta Rühlmann, Andreas Maercker, Birgit Kleim

**Affiliations:** 1 Department of Psychology, University of Zurich, Binzmühlestrasse 14/17, 8050 Zurich, Switzerland; 2 Department of Psychiatry, Psychotherapy and Psychosomatics, Psychiatric Hospital, University of Zurich, Zurich, Switzerland; 3 Department of Experimental Psychopathology and Psychotherapy, Psychiatric University Hospital, University of Zurich, Lenggstrasse 32, 8032 Zurich, Switzerland

**Keywords:** Sleep, Loneliness, Social connectedness, Diary, Wearables, Psychology, Human behaviour

## Abstract

Greater loneliness as well as a lack of social connectedness have often been associated with poorer sleep. However, the temporal dynamics and direction of these associations remain unclear. Aim of the current study was to examine bi-directional associations between loneliness/social connectedness and sleep in 48 stress-exposed medical students during their first medical internship, considered a period of heightened stress. We obtained trait-level questionnaire data on loneliness and global sleep completed before and during the internship as well as state-level diary- and wearable-based data on daily changes in social connectedness and sleep collected twice over the period of seven consecutive days, once before and once during the internship. Bi-directional associations among greater loneliness and higher daytime dysfunction on trait-level were identified. In addition, several uni-directional associations between loneliness/social connectedness and sleep were found on trait- and state-level. In sum, findings of this study point at a bi-directional relation among loneliness/social connectedness and sleep, in which variables seem to reciprocally influence each other across longer-term periods as well as on a day-to-day basis.

## Introduction

Sleep and loneliness are key determinants of physical and mental health, both related to a number of negative health outcomes including cognitive decline, cardiovascular diseases, depression, suicidal behavior, and mortality^1–10^. Around 30–40% of the general population suffer from sleep difficulties^11–14^ and around 10% from loneliness^15^. Loneliness is typically defined as a perceived discrepancy between an individual’s desired and actual social relations^16^, which can lead to the painful experience that one does not belong^17^. Social connectedness is often described as an opposing construct to loneliness thought to simply reflect the other end of the continuum. However, more recently, researchers have turned to consider indicators of social connectedness and loneliness as related but distinct and not symmetrically opposite concepts^18^. Indeed, previous research has also pointed at differences in their impact on health, such that for example low social support, but not loneliness, was found to significantly predict cardiovascular diseases^19^; findings that highlight the need to investigate these constructs simultaneously but separately.

A recently published meta-analysis by Hom and colleagues^20^ confirmed robust cross-sectional associations between loneliness and overall sleep disturbances as well as specific sleep problems (i.e., nightmares, poor sleep quality, insomnia). Similarly, another meta-analysis reported a medium-sized correlation between loneliness and self-reported sleep disturbance^21^. With regard to social connectedness, a systematic review of Gordon and colleagues^22^ pointed at significant associations between sleep and social relationships, such that high quality relationships were found to be linked to better sleep, while low quality relationships were found to be associated with sleep difficulties. Likewise, a meta-analysis by Kent de Grey and colleagues^23^ revealed robust associations between greater social support and better sleep. Interestingly, previous research suggests that, generally, associations among sleep and affective variables might be particularly pronounced in the context of stress [e.g.,^24,25^]. Given the negative consequences of sleep and loneliness/lack of social connectedness, a better understanding of the interplay among these variables, and particularly the temporal dynamics and direction of associations, is crucial.

From a theoretical perspective, some have argued that given the human need to rely on others in order to survive, the feeling of loneliness is comparable to feelings of threat or vulnerability, which may result in poor sleep quality via changes in hypervigilance^26–28^. On the other hand, it has been noted, that sleep disturbances may lead to difficulties in emotion regulation contributing to the perception of feeling lonely and unconnected^1,20^. Empirical findings based on longitudinal designs point at a bi-directional relation among loneliness/social connectedness and sleep, such that higher levels of loneliness and lower levels of social connectedness predict more disturbed sleep, and vice versa [e.g.,^20,22^]. However, these findings have to be interpreted with caution as the number of longitudinal studies is limited. In addition, current understanding of these longitudinal associations is primarily based on studies investigating the effects across multi-month time frames, i.e. predicting sleep based on loneliness assessed several months earlier, or vice versa. However, since loneliness, social connectedness and sleep vary within individuals on a day-to-day basis, intensive longitudinal designs in daily life are needed in order to shed light on the interplay among these variables and to understand the temporal dynamics of these associations.

To date, a handful of studies have examined associations among loneliness/social connectedness and sleep on a daily basis. In a 14-days daily experience study of 78 individuals in romantic relationships, Gordon and Chen^29^ found that poor sleep was a significant predictor of more relationship conflicts. Investigating the opposite direction in a 14-days ecological momentary assessment (EMA) study of 221 Latino/a college students, Hussain and colleagues^30^ found that on days that students felt lonelier than they usually do, they indicated poorer overall sleep quality the next morning. The effect of loneliness on self-reported total sleep time was nonsignificant. In a similar daily diary study by Tighe and colleagues^31^ conducted in a sample of 50 younger and 48 older community dwelling adults, a significant negative association was found between the frequency of alone activities and sleep duration in older individuals. However, all of the aforementioned studies only tested one direction of the association and the sleep parameters used were based on self-reports. Using objective estimates of sleep and investigating the influence of loneliness on subsequent sleep, Sladek and Doane^32^ found that within-person increases in daily social connection significantly predicted longer time spent in bed and more actual time asleep that night, but only for students high on trait-loneliness. In those low on trait-loneliness increases in daily social connection predicted higher sleep onset latency.

To the best of our knowledge, so far, three studies have investigated bi-directional associations among loneliness, social connectedness and sleep on a daily-level. In a three-day diary-study of 215 adults, Hawkley et al.^33^ found that daily variations in loneliness were a significant predictor of subsequent feelings of daytime dysfunction, which the authors report as indicative of poor sleep quality, and vice versa. The influence of feelings of loneliness on daytime dysfunction was independent of differences in sleep duration. However, no objective sleep measures were used in this study. Similarly, in a micro-longitudinal study covering two consecutive nights and their two subsequent days, Ben Simon and Walker^34^ observed a negative bi-directional association between decreased sleep efficiency and increased loneliness levels. Extending the assessment period to three weeks in a sample of 641 working adults, Holding and colleagues^35^ found higher levels of sleepiness to be a significant predictor of a lower probability and duration of social activity. Additionally, a long duration of social activity (> five hours) predicted a reduced sleep time. However, all of these studies did not include any objective sleep measures.

Based on these findings, sleep may be bi-directionally associated with loneliness and social connectedness on a daily-level pointing at a downward spiral where variables continue to negatively influence each other, but associations may be complex and may also differ across sleep measurement (self-report vs. objective measures) and sleep indices (e.g., total sleep time, daytime dysfunction, sleep onset latency). Although differences have been identified among self-reported and objectively measured sleep indices^36^, both are increasingly recognized as representing unique features of sleep and its related experiences^37^.

The current study aimed to contribute to a better understanding of the temporal dynamics and the direction of the association between sleep and loneliness/social connectedness. As associations among sleep and affective variables might be particularly pronounced in the context of stress [e.g.,^24,25^], we examined associations in a sample of stress-exposed individuals, medical students during their first medical internship. The present study was composed of two parts, trait-level and state-level analyses, respectively. For the trait-level part, we collected questionnaire data on trait-level loneliness and sleep before and during the internship and were interested in associations between loneliness and global sleep. For state-level analyses, we used diary- and wearable-based data on social connectedness and sleep assessed twice over the period of seven consecutive days, once collected before the start of the internship and once during the internship (each time covering a seven-day period). Here, we were particularly interested in associations between daily changes in sleep and social connectedness. Social connectedness indicators included social safeness, amount of time spent with family and friends, and the number of daytime positive social interactions. Sleep indices were composed of restfulness of sleep, total sleep time, sleep onset latency, wake-up frequency, and nocturnal heart rate (as a measure of nocturnal arousal indicative of stress-related sleep disturbances, see also [e.g.,^38,39^]). To the best of our knowledge, this study is the first to examine such bi-directional associations on a day-to-day basis using both objective as well as self-reported sleep indices.

*On trait-level*, based on self-report questionnaires, we hypothesized that, greater loneliness at baseline (prior to the start of the internship) will predict more disturbed sleep three months later (midterm of the internship), and that more disturbed sleep at baseline (prior to the start of the internship), in turn, will predict greater loneliness three months later (midterm of the internship). *On state-level*, i.e., based on diary and wearable assessments, we hypothesized that greater social connectedness will predict less disturbed sleep the following night in a time-lagged fashion, and that less disturbed sleep, in turn, will predict greater social connectedness the next day.

## Methods

The present report is part of a larger study on sleep and mental health in medical students. Details of the study procedure have been described previously [e.g.,^40^].

### Participants

We recruited 50 medical students of the University of Zurich using advertisements sent via emails, flyers posted in the Zurich area, and presentations in lectures. They were enrolled in their fifth year of medical training and just about to begin their first medical internship, which has been described as a real-world stressor^41–43^. Two of these participants did not complete baseline self-report questionnaires, resulting in a final sample of 48 individuals.

### Study design and procedure

**Figure 1:**
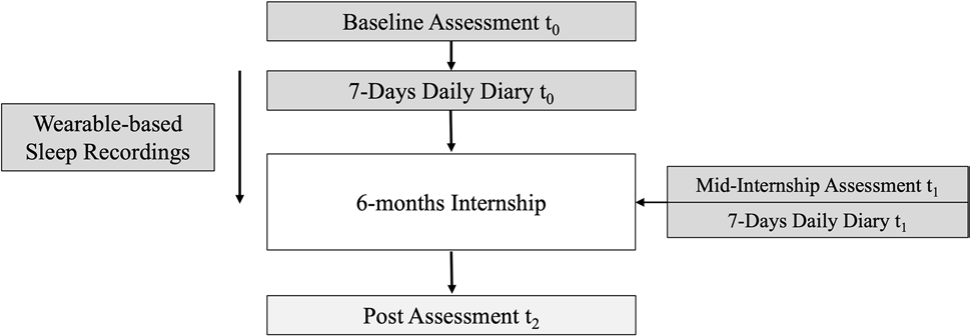
Study design. *Note.* Dark grey highlighted assessments were part of the current investigation.

As part of a larger set of projects, medical students were invited to an initial lab session prior to the start of their internship during which they were screened for symptoms of depression and psychosis, suicidality, and sleep disorders or disturbances by means of structured diagnostic interviews and questionnaires. Participants who were found to be eligible and had provided informed consent were asked to complete the first questionnaire battery, assessing demographic characteristics, current stress load, emotional state, personality, anxiety, depressive symptoms, loneliness and sleep. For objective sleep recordings, participants also received a FitbitChargeHR™ (Fitbit Inc., San Francisco, CA, USA), which they were asked to wear continuously on the wrist over the three month period. One week prior to the start of their internship, students were asked to complete a diary over the period of seven consecutive days (t_0_) consisting of two parts, an evening and a morning section. In the evening section, students were asked to report on their day and well-being, including measures of social connectedness. In the morning section, they were asked to report on their sleep. Three months after the start of their internships (t_1_), the same questionnaires were administered as well as a second diary (again over the period of seven consecutive days). At six months, post- internship (t_2_), participants were asked to complete the same questionnaires, but no diary was administered. At the end of the study, participating students were debriefed as well as reimbursed for their time. The study was approved by the local ethics review board at the University of Zurich and was conducted in accordance with all relevant guidelines and regulations. Figure 1 depicts the study design.

For this study, we originally decided to focus on time point t_1_ using questionnaire data collected before (t_0_) and during internship (t_1_) as well as diary- and wearable-based data collected during the internship (t_1_), as we thought that this time point may constitute a peak period of stress during the internship given the greater independence and responsibility students have to assume [e.g.,^44,45^]. However, since analyses revealed that trait-level stress levels did actually not significantly differ from t_0_ to t_1_, we decided to add diary- and wearable-based data collected prior to the internship (t_0_) to the current manuscript.

### Measures

#### Trait-level part: self-report questionnaires (t_0_ and t_1_)

##### Loneliness

*Loneliness* was measured with the Three-Item Loneliness Scale^46^. The scale encompasses three items, such as *how often do you feel that you lack companionship?.* Items were rated on a 3-point scale ranging from (1) *hardly ever* to (3) *often* and were then added together to a loneliness sum score. A higher score indicates more loneliness. Cronbach’s alpha was 0.66 at t_0_ and 0.64 at t_1_. The alpha for the Three-Item Loneliness Scale has been reported to be somewhat smaller than the ones typically found for the full scale^46^. However, it still has been recommended as a robust and reliable instrument for assessing general feelings of loneliness^46^.

##### Sleep quality and disturbances

*Sleep quality and disturbances* were assessed with the Pittsburgh Sleep Quality Index [PSQI,^47^], which is a scale measuring sleep quality and disturbances within the last month. The questionnaire contains nineteen self-rated items as well as five items rated by the roommate or bed partner (if available). In this study, we focused on the self-rated questions, which generate seven component scores: Subjective sleep quality, sleep onset latency, sleep duration, habitual sleep efficiency, sleep disturbances, use of sleeping medication, and daytime dysfunction. The sum of these seven component scores yields a global sleep quality score. Our analyses focused on *sleep duration*, *sleep latency*, *subjective sleep quality*, *daytime dysfunction* (e.g., *During the past month, how often have you had trouble staying awake while driving, eating meals, or engaging in social activity?*), and the *global PSQI score*. The type of answer scale varied depending on the item used. Each component score had a range from 0 to 3 points with a score of “0” indicating no difficulty and a score of “3” indicating severe difficulty. The global score had a range of 0–21 points with a score of “0” indicating no difficulty and “21” indicating severe difficulty in all areas.

#### State-level part: diary

Participants filled in a paper–pencil based diary over the period of seven consecutive days, both before (t_0_) and during the internship (t_1_). Participant were asked to complete the evening section just prior to turning off the lights and the morning section just after waking up. The diary measured indicators of social connectedness as part of the evening section(daily social safeness, number of daytime positive social interactions, amount of time spent with family and friends) as well as different sleep indices as part of the morning section.

#### Social connectedness indicators

##### Social Safeness

*Social Safeness* was measured using four items each evening. Selected items of the Social Safeness and Pleasure Scale^48^ were used, such as *I feel a sense of belonging* or *I feel secure and wanted*. The wording of the answer scale was slightly adapted and the scale ranged from (1) *does not apply* to (5) *fully applies* (original wording: (1) *almost never* to (5) *almost all of the time*). Mean scores were calculated. A higher score indicates more social safeness. Cronbach’s alpha was 0.86 at t_0_ and 0.90 at t_1_.

##### Number of daytime positive social interactions (positive interactions)

*Number of Daytime Positive Social Interactions* was measured with one item each evening based on Sladek and Doan^32^ (*How many positive interactions did you have today (e.g., supportive, enjoyable, rewarding, affirming)?*)*.* The answer scale ranged from (0) *one* to (1) *several* to (2) *many*.

##### Amount of time spent with family and friends (social time)

*Amount of Time spent with Family and Friends (Social Time)* was measured with one item each evening based on Sladek and Doan^32^. The original item asked participants to indicate how much time they spent today with others (*How much time did you spend today with others, talking or listening to them*?). For the present study, the item was slightly adapted in order to take into account the context of a full-time internship and was therefor changed to: *How much of your leisure time today have you spent talking to or listening to other people outside your work environment (i.e. partner, family members, friends, acquaintances)?* The answer scale ranged from (0) *none or little time* to (4) *most of the day or all day*.

##### Subjective sleep

*Subjective sleep* was measured using four items. Each morning, participants were asked to indicate their *subjective restfulness of sleep* (*How restful was your sleep?*) on a scale ranging from (0) *not restful at all* to (4) *very restful*, their *subjective total sleep time (TST)* in hours (*How long did you sleep in total?*), their *subjective sleep onset latency (SOL)* in minutes (*How long did it take for you to fall asleep after turning off the lights?*) and their *subjective wake-up frequency* (*How often did you wake up during the night?*). A weighted s*ubjective wake-up index (WUI)* was created for each night (dividing the number of awakenings by total sleep time).

#### State-level part: daily objective sleep-recordings derived from wearables

The following objective sleep measures were derived from the FitbitChargeHR™: SOL (in minutes), TST (in minutes), nocturnal heart rate (HR) in beats per minute (bpm), and wake-up frequency (and WUI). Data on nocturnal heart rate were only available from the start of the internship and, thus, only included in state-level analyses at t_1_, but not t_0_. Diary falling asleep times were compared with those of wearable recordings for each day and those wearable recordings that indicated sleep more than 15 min before the diary sleep time were excluded. This resulted in the exclusion of 11 single recordings out of a total of 245 collected objective recordings at t_0_, and in the exclusion of 17 single recordings out of a total of 252 objective recordings at t_1_. In addition, at t_0_, three single wearable recordings were excluded given that an extreme difference to diary-based falling asleep times was observed (e.g., diary-based bedtime in the night and wearbale-based start time in the afternoon). In a previous study, recordings of the FitbitChargeHR™ demonstrated good agreement with electrocardiography and polysomnography measures, as for example shown by high overall accuracy (91%) and high sensitivity rates (97%)^49^.

#### Demographic information and control variables

Participants were asked to fill in a sociodemographic questionnaire assessing basic demographic information, such as age, sex, relationship status and housing situation (living alone vs. with others). In addition, we assessed stress as a control variable using the Perceived Stress Scale [PSS-4, ^50^] as a trait-level measure and a standard visual analog stress scale as a state-level measure as part of the evening section of the diary (item: *How stressful was your day?*; visual analog answer scale ranging from 0 to 100).

### Data analysis

Data were analyzed using the statistic software R Version 3.5.1^51^. To examine bi-directional associations among sleep and loneliness on trait-level, prospective linear regression models were estimated using the R package *stats*^51^. The models were prospective in the sense that the independent variable was measured before the dependent variable. We ran different prospective models predicting sleep/loneliness at t_1_ from loneliness/sleep at t_0_. Baseline (t_0_) loneliness/sleep, stress (at t_0_ and at t_1_), age, sex, relationship status (single vs. in a relationship) as well as housing situation (living alone vs. with others) were entered into the models as control variables. These final models were then used to test our hypotheses.

We estimated multilevel models (hierarchical linear models, HLM) to examine bi-directional assocations among sleep and social connectedness indicators (social safeness, social time, positive interactions) on state-level and to account for the nesting of assessments within participants and across time. We calculated intraclass correlations (ICC) to assess the ratio of between-person variance to the total variance (between and within variance). The R package *nlme*^52^ was used. Predictors without meaningful zero-points (stress, social safeness, social time, positive interactions, restfulness of sleep) as well as age were grand-mean-centered to make interpretation of intercepts clearer. We tested all associations prospectively (i.e., the independent variable was measured before the dependent variable) and controlled for lagged outcomes to determine directionality. For example, in the first model, predicting restfulness of sleep from social safeness, we added the previous night’s restfulness of sleep into the model to make sure that whatever effect we found for social safeness, would not include variance that is due to yesterday’s restfulness of sleep and its effect on social safeness (or directly on today’s restfulness of sleep). We followed the following four steps to build a final model: (1) we created a model comprising our main predictors (social connectedness/sleep, lagged outcome), (2) tested whether a random intercept or random intercept and slopes model matches the data significantly better, (3) tested whether including autocorrelation fits the data better and (4) added control variables to the model such as age, sex, stress, measurement time, relationship status and housing situation (living with others vs. alone). This final model was then used to test our hypotheses. Characteristics of each final model are indicated in the results tables. All data and code are available on OSF (https://osf.io/e5awd/?view_only=b4122f52eb8846488454473c0be457d5).

## Results

### Descriptive statistics

From the overall sample, 48 participants completed self-report questionnaires at both t_0_ and t_1_ (trait-level). All of these participants completed diary entries at t_0_ and 46 completed diary entries at t_1_ (state-level). Objective sleep reordings were collected from 35 participants during diary week at t_0_ and from 36 individuals during diary week at t_1_. Sample characteristics are summarized in Table 1, distribution of trait-level study variables in Table 2 and distribution of state-level study variables in Table 3. Trait-level stress levels did not statistically significantly differ from t_0_ to t_1_ (*t*(93) = − 0.05, *p* = 0.961). Since only one participant lived alone, the control variable housing situation was excluded from our analyses. Wearable-based SOL could not reliably be indexed due to missing data on the “lights off” index for most nights. Thus, it could not reliably be distinguished between zero or missing wearable-based SOL values. As a result, wearable-based SOL was excluded from our analyses.

**Table 1:** Sample demographic characteristics (n = 48).

		M	SD
Age		23.60	1.28

		N	%
Sex	Female	36	75.00
	Male	12	25.00
Ethnicity	Swiss	47	97.92
	Other European country	1	2.08
Relationship status	Single	15	31.25
	In relationship	33	68.75
Housing situation	Living alone	1	2.08
	Living with others	47	97.92

**Table 2:** Distribution of trait-level study variables (n = 48).

		M	SD	Min	Max
Self-report questionnaires t_0_	Loneliness	4.42	1.23	3	9
	Sleep quality	0.81	0.49	0	2
	Sleep latency	0.98	0.76	0	3
	Sleep duration	0.10	0.37	0	2
	Daytime dysfunction	0.73	0.64	0	2
	Global PSQI score	4.17	1.69	0	9
	Stress	4.79	1.99	1	9
Self-report questionnaires t_1_	Loneliness	4.50	1.35	3	8
	Sleep quality	0.98	0.39	0	2
	Sleep latency	0.65	0.60	0	2
	Sleep duration	0.33	0.48	0	1
	Daytime dysfunction	0.96	0.77	0	3
	Global PSQI score	4.04	1.38	1	7
	Stress	4.81	2.17	1	9

**Table 3:** Distribution of state-level study variables at t_0_ and t_1_.

			M	SD	Min	Max
T_0_ (n = 48)	Diary	Social safeness	4.26	0.68	1.50	5
		Social time	2.74	1.29	0	4
		Positive interactions	1.30	0.61	0	2
		Stress	34.65	28.91	0	100
		Restfulness of sleep	2.70	0.83	0	4
		TST (hrs.)	7.51	1.25	2	12
		SOL (min.)	12.57	17.57	0	180
		No. awakenings	0.97	1.37	0	10
		WUI	0.14	0.25	0	2.50
	Wearable	TST (hrs.)	6.89	1.36	0.78	10
		No. awakenings	13.99	7.41	1	44
		WUI	2.03	0.97	0.12	5.56
T_1_ (n = 46)	Diary	Social safeness	4.01	0.84	1.25	5
		Social time	2.10	1.40	0	6
		Positive interactions	1.31	0.66	0	2
		Stress	32.24	25.48	0	100
		Restfulness of sleep	2.62	0.82	0	4
		TST (hrs.)	7.34	1.26	3.50	12
		SOL (min.)	8.33	10.98	0	70
		No. awakenings	0.77	1.17	0	9
		WUI	0.11	0.19	0	1.71
	Wearable	TST (hrs.)	6.90	1.28	3.70	11.27
		No. awakenings	12.96	7.30	0	43
		WUI	1.88	0.99	0	5.97
		Nocturnal HR	62.25	9.22	39.95	92.57

*Note.* Social time,  amount of time spent with family and friends; positive interactions, number of daytime positive social interactions; TST, total sleep time; SOL, sleep onset latency; WUI, wake-up index (no. awakenings/TST); HR, heart rate.

### Trait-level analyses

All models reported are those including control variables, as these were the models that were used to test our hypotheses (see data analysis section).

#### Effect of loneliness on sleep

**Figure 2:**
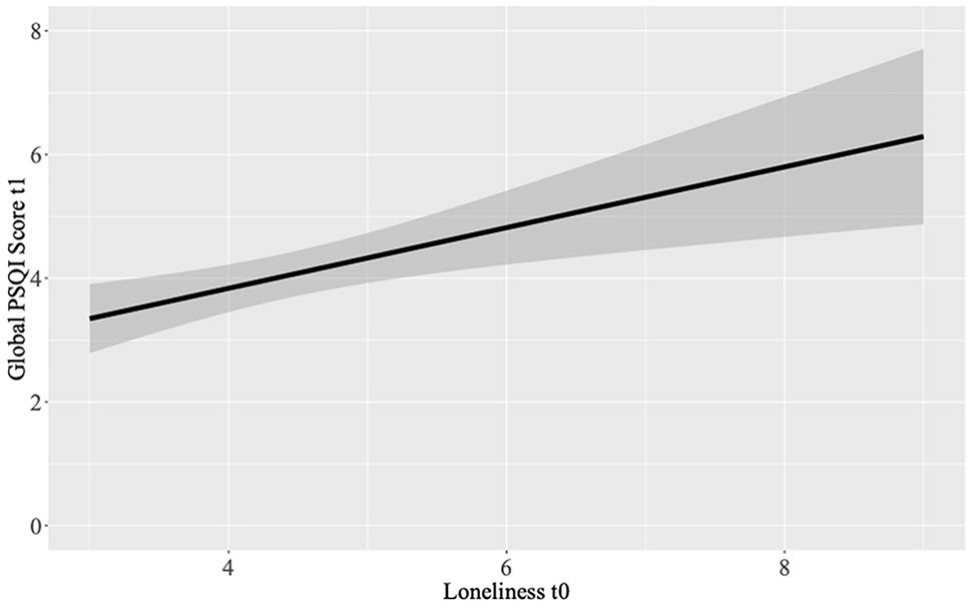
Prediction of global PSQI score t_1_ from loneliness t_0_ (trait-level analyses).

Greater loneliness at t_0_ statistically significantly predicted higher daytime dysfunction (B = 0.276, SE = 0.102, p = 0.010) as well as poorer global sleep quality (global PSQI score) at t_1_ (B = 0.377, SE = 0.175, p = 0.037). None of the other regression models were significant (all *p* > 0.142). See Table 4 for the results and Fig. 2 for a depiction of the statistically significant effect of loneliness at t_0_ on global sleep quality at t_1_.

**Table 4:** Trait-level analyses predicting sleep t_1_ from loneliness t_0_ (n = 48).

Outcome (t_1_)	Predictor (t_0_)	B (SE)	95% CI	p
Sleep quality	Loneliness	− 0.013 (0.058)	[− 0.130, 0.105]	0.830
Sleep latency		0.117 (0.078)	[− 0.041, 0.276]	0.143
Sleep duration		0.041 (0.063)	[− 0.087, 0.168]	0.525
Daytime dysfunction		0.276 (0.102)	[0.070, 0.481]	0.010*
Global PSQI Score		0.377 (0.175)	[0.024, 0.730]	0.037*

*Note.* B, regression coefficient; SE, standard error; CI, confidence interval; * = p < 0.05, ** = p < 0.01, *** = p < 0.001.

#### Effect of sleep on loneliness

**Figure 3:**
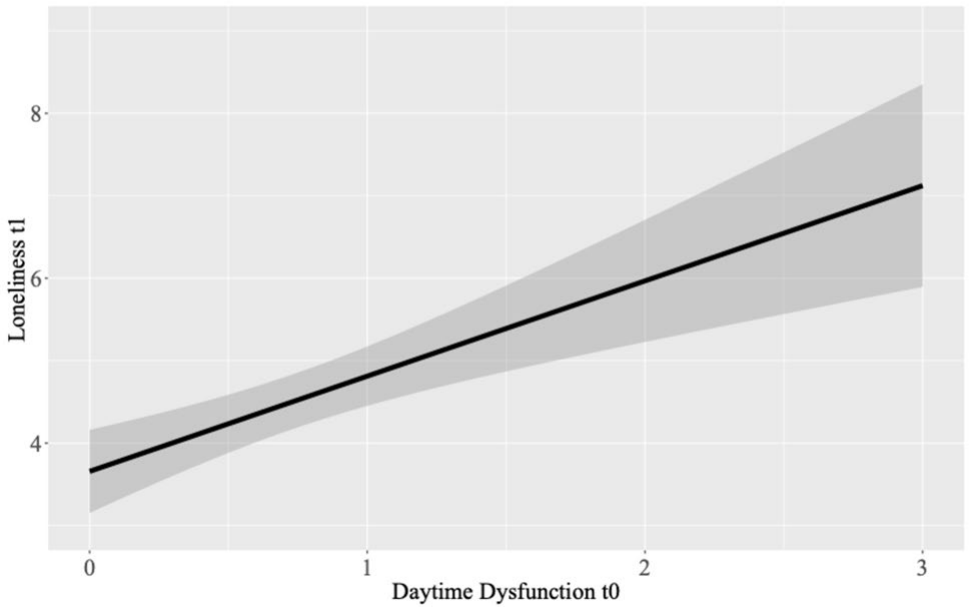
Prediction of loneliness t_1_ from daytime dysfunction t_0_ (trait-level analyses).

The effect of daytime dysfunction at t_0_ on loneliness at t_1_ was at the threshold of statistical significance, such that results indicated a trend towards higher daytime dysfunction being associated with greater loneliness (B = 0.531, SE = 0.263, p = 0.050); see Fig. 3. The remaining regression models were all nonsignificant (all *p* > 0.266). See Table 5 for the results.

**Table 5:** Trait-level analyses predicting loneliness t_1_ from sleep t_0_ (n = 48).

Outcome (t_1_)	Predictors (t_0_)	B (SE)	95% CI	p
Loneliness	Sleep quality	0.106 (0.296)	[− 0.492, 0.704]	0.722
	Sleep latency	0.033 (0.195)	[− 0.362, 0.428]	0.866
	Sleep duration	0.062 (0.400)	[− 0.746, 0.870]	0.877
	Daytime dysfunction	0.531 (0.263)	[0.000, 1.062]	0.050
	Global PSQI Score	0.096 (0.086)	[− 0.077, 0.270]	0.267

*Note.* B, regression coefficient; SE, standard error; CI, confidence interval; * = p < 0.05, ** = p < 0.01, *** = p < 0.001.

### State-level analyses

Calculation of ICCs for all state variables (diary- and wearable-based data) indicated that these had sufficient within-person variability. ICC scores at t_0_ were 0.52 for social safeness, 0.30 for social time, 0.25 for positive interactions, 0.39 for stress, 0.15 for restfulness of sleep, 0.18 for self-reported TST, 0.22 for self-reported SOL, 0.22 for self-reported WUI, 0.17 for wearable-based TST and 0.38 for wearable-based WUI. ICC scores at t_1_ were 0.52 for social safeness, 0.19 for social time, 0.39 for positive interactions, 0.28 for stress, 0.12 for restfulness of sleep, 0.17 for self-reported TST, 0.45 for self-reported SOL, 0.24 for self-reported WUI, 0.16 for wearable-based TST, 0.60 for wearable-based WUI and 0.64 for nocturnal HR.

All models reported are those including control variables, as these were the models that were used to test our hypotheses (see data analysis section).

### Analyses t_0_

#### Effect of daily changes in social connectedness on sleep

**Figure 4:**
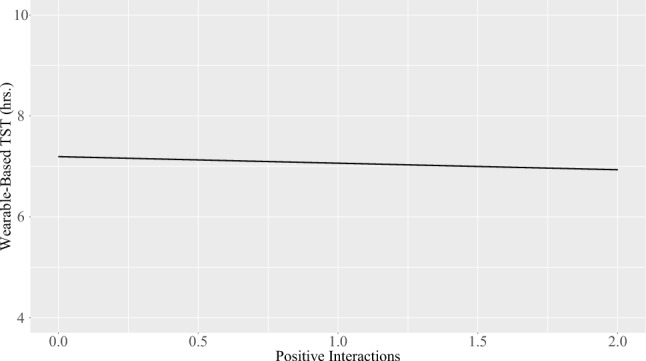
Prediction of wearable-based TST from number of positive social interactions (state-level analyses t_0_). *Note.* X-Axis values for positive interactions refer to the original (i.e., non-mean-centered) scores.

A higher number of positive social interactions statistically significantly predicted a shorter wearable-based TST (B = − 0.327, SE = 0.164, p = 0.048); see Fig. 4. The remaining models were all nonsignificant (all *p* > 0.108). See Table 6 for the results, and the supplementary material for the outcomes of the different versions of the models and the full final results table including the effect of stress (Tables S1 and S2).

**Table 6:** State-level analyses predicting sleep from social connectedness at t_0_ (n = 48).

Outcome	Predictors	B (SE)	95% CI	p
Diary				
Restfulness of sleep	Social safeness^a^	0.126 (0.078)	[− 0.028, 0.279]	0.109
	Social Time^a^	0.027 (0.044)	[− 0.059, 0.113]	0.536
	Positive interactions^a^	0.131 (0.084)	[− 0.034, 0.296]	0.120
TST (hrs.)	Social safeness^a^	0.154 (0.123)	[− 0.090, 0.397]	0.215
	Social time^a^	0.080 (0.066)	[− 0.050, 0.210]	0.225
	Positive interactions^a^	0.055 (0.127)	[− 0.196, 0.305]	0.668
SOL (min.)	Social safeness^bc^	− 3.369 (3.415)	[− 10.096, 3.358]	0.325
	Social time^ac^	− 1.171 (0.924)	[− 2.995, 0.652]	0.207
	Positive interactions^a^	− 2.745 (1.793)	[− 6.277, 0.788]	0.127
WUI	Social safeness^a^	0.017 (0.026)	[− 0.034, 0.068]	0.514
	Social time^a^	0.006 (0.015)	[− 0.023, 0.036]	0.677
	Positive interactions^b^	− 0.039 (0.036)	[− 0.110, 0.031]	0.273
Wearable				
TST (hrs.)	Social safeness^bc^	− 0.269 (0.169)	[− 0.602, 0.065]	0.114
	Social time^a^	− 0.048 (0.090)	[− 0.226, 0.130]	0.596
	Positive interactions^ac^	− 0.327 (0.164)	[− 0.651, − 0.004]	0.048*
WUI	Social safeness^ac^	0.160 (0.111)	[− 0.059, 0.379]	0.152
	Social time^a^	0.036 (0.060)	[− 0.083, 0.156]	0.550
	Positive interactions^ac^	− 0.173 (0.118)	[− 0.407, 0.060]	0.145

*Note.* TST, total sleep time; SOL, sleep onset latency; WUI, wake-up index (no. awakenings/TST); HR, heart rate; social time, amount of time spent with family/friends; positive interactions, number of daytime positive social interactions; B, regression coefficient for the fixed effect; SE, standard error; CI, confidence interval; * = p < 0.05, ** = p < 0.01, *** = p < 0.001, grand-mean-centered scores were used for social safeness, positive interactions and social time, ^a^random intercept model, ^b^random intercept and slopes model, ^c^model includes autocorrelation.

#### Effect of daily changes in sleep on social connectedness

**Figure 5:**
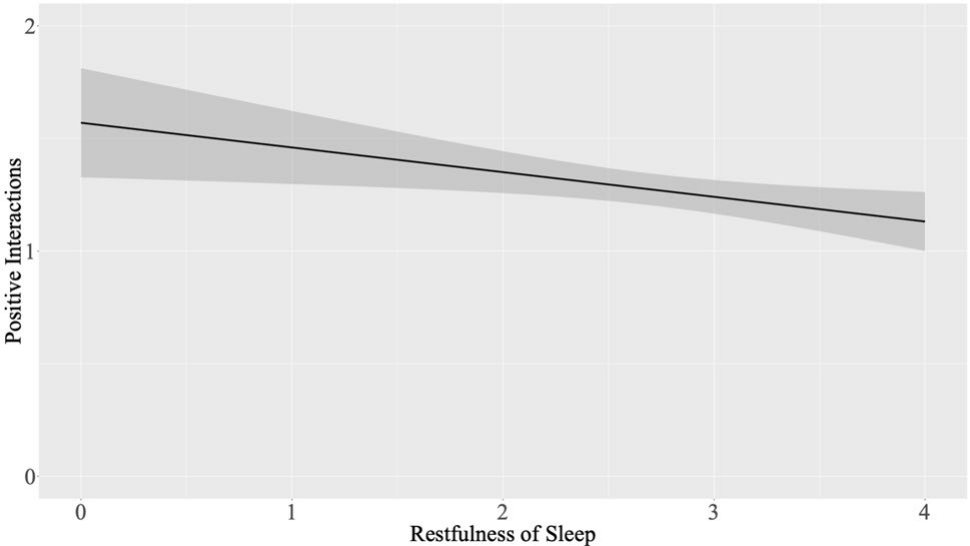
Prediction of number of positive social interactions from restfulness of sleep (state-level analyses t_0_). *Note.* X-Axis values for restfulness of sleep refer to the original (i.e., non-mean-centered) scores.

A longer wearable-based TST statistically significantly predicted less social safeness (B = − 0.068, SE = 0.031, p = 0.031). In addition, greater restfulness of sleep statistically significantly predicted a smaller number of positive social interactions (B = − 0.117, SE = 0.042, p = 0.006); see Fig. 5. All remaining models were nonsignificant (all p > 0.091). See Table 7 for the results, and the supplementary material for the outcomes of the different versions of the models and the full final results table including the effect of stress (Tables S3 and S4).

**Table 7:** State-level analyses predicting social connectedness from sleep at t_0_ (n = 48).

Outcome	Predictors	B (SE)	95% CI	p
Social safeness	Diary			
	Restfulness of sleep^ac^	− 0.015 (0.040)	[− 0.094, 0.063]	0.700
	TST (hrs.)^ac^	− 0.032 (0.028)	[− 0.086, 0.023]	0.251
	SOL (min.)^ac^	0.001 (0.002)	[− 0.002, 0.005]	0.460
	WUI^a^	− 0.058 (0.154)	[− 0.361, 0.245]	0.706
	Wearable			
	TST (hrs.)^ac^	− 0.068 (0.031)	[− 0.130, − 0.006]	0.031*
	WUI^ac^	− 0.045 (0.048)	[− 0.140, 0.049]	0.345
Social time	Diary			
	Restfulness of sleep^a^	− 0.050 (0.106)	[− 0.258, 0.159]	0.640
	TST (hrs.)^a^	0.028 (0.068)	[− 0.107, 0.164]	0.680
	SOL (min)^a^	0.007 (0.004)	[− 0.001, 0.016]	0.105
	WUI^ac^	0.194 (0.389)	[− 0.577, 0.966]	0.619
	Wearable			
	TST (hrs.)^a^	− 0.021 (0.077)	[− 0.175, 0.132]	0.783
	WUI^a^	0.034 (0.118)	[− 0.200, 0.268]	0.773
Positive interactions	Diary			
	Restfulness of sleep^a^	− 0.117 (0.042)	[− 0.201, − 0.034]	0.006**
	TST (hrs.)^a^	− 0.027 (0.029)	[− 0.085, 0.031]	0.364
	SOL (min.)^a^	0.001 (0.002)	[− 0.003, 0.004]	0.787
	WUI^a^	0.281 (0.166)	[− 0.046, 0.608]	0.092
	Wearable			
	TST (hrs.)^a^	− 0.030 (0.033)	[− 0.095, 0.035]	0.358
	WUI^a^	− 0.015 (0.049)	[− 0.112, 0.082]	0.755

*Note.* TST, total sleep time; SOL, sleep onset latency; WUI, wake-up index (no. awakenings/TST); HR, heart rate; social time, amount of time spent with family/friends; positive interactions, number of daytime positive social interactions; B, regression coefficient for the fixed effect; SE, standard error; CI, confidence interval; * = p < 0.05, ** = p < 0.01, *** = p < 0.001, grand-mean-centered scores were used for self-reported restfulness of sleep, ^a^random intercept model, ^b^random intercept and slopes model, ^c^model includes autocorrelation.

### Analyses t_1_

#### Effect of daily changes in social connectedness on sleep

**Figure 6:**
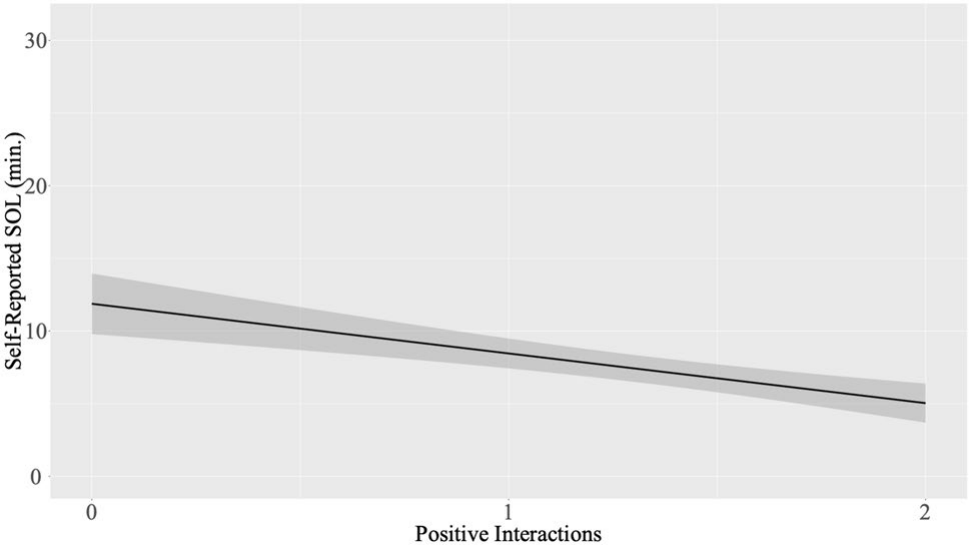
Predicting self-reported sleep onset latency from number of positive social interactions (state-level analyses t_1_). *Note.* X-Axis values for positive interactions refer to the original (i.e., non-mean-centered) scores.

Higher social safeness statistically significantly predicted greater restfulness of sleep (B = 0.128, SE = 0.059, p = 0.031). Further, a higher number of positive social interactions was a statistically significant predictor of shorter self-reported SOL (B = − 4.324, SE = 1.379, p = 0.002) and lower wearable-based WUI (B = − 0.280, SE = 0.100, p = 0.006). The remaining models were all nonsignificant (all *p* > 0.050). See Table 8 for the results and Fig. 6 for a depiction of the statistically significant effect of positive interactions on self-reported SOL. Tables S5 and S6 in the supplementary material show the outcomes of the different versions of the models as well as the full final results table including the effect of stress.

**Table 8:** State-level analyses predicting sleep from social connectedness at t_1_ (n = 46).

Outcome	Predictors	B (SE)	95% CI	p
Diary				
Restfulness of sleep	Social safeness^a^	0.128 (0.059)	[0.012, 0.244]	0.031*
	Social Time^a^	0.045 (0.041)	[− 0.036, 0.127]	0.273
	Positive interactions^a^	0.047 (0.079)	[− 0.109, 0.203]	0.553
TST (hrs.)	Social safeness^ac^	− 0.026 (0.096)	[− 0.216, 0.164]	0.789
	Social time^ac^	− 0.125 (0.064)	[− 0.250, 0.000]	0.051
	Positive interactions^ac^	− 0.026 (0.130)	[− 0.282, 0.231]	0.845
SOL (min.)	Social safeness^a^	− 1.252 (0.784)	[− 2.797, 0.293]	0.112
	Social time^a^	− 0.758 (0.488)	[− 1.720, 0.204]	0.122
	Positive interactions^b^	− 4.324 (1.379)	[− 7.042, − 1.607]	0.002**
WUI	Social safeness^a^	0.023 (0.016)	[− 0.008, 0.055]	0.146
	Social time^ac^	− 0.003 (0.010)	[− 0.022, 0.016]	0.756
	Positive interactions^ac^	0.002 (0.020)	[− 0.037, 0.041]	0.924
Wearable				
TST (hrs.)	Social safeness^a^	− 0.154 (0.136)	[− 0.424, 0.116]	0.262
	Social time^a^	0.039 (0.086)	[− 0.132, 0.209]	0.656
	Positive interactions^a^	0.097 (0.179)	[− 0.256, 0.451]	0.587
WUI	Social safeness^ac^	− 0.083 (0.066)	[− 0.213, 0.047]	0.210
	Social time^ac^	− 0.091 (0.049)	[− 0.188, 0.006]	0.066
	Positive interactions^ac^	− 0.280 (0.100)	[− 0.478, − 0.081]	0.006**
Nocturnal HR	Social safeness^ac^	0.253 (0.573)	[− 0.881, 1.388]	0.659
	Social time^ac^	0.238 (0.411)	[− 0.576, 1.051]	0.564
	Positive interactions^ac^	− 0.025 (0.801)	[− 1.612, 1.562]	0.976

*Note.* TST, total sleep time; SOL, sleep onset latency; WUI, wake-up index (no. awakenings/TST); HR, heart rate; social time, amount of time spent with family/friends; positive interactions, number of daytime positive social interactions; B, regression coefficient for the fixed effect; SE, standard error; CI, confidence interval; * = p < 0.05, ** = p < 0.01, *** = p < 0.001, grand-mean-centered scores were used for social safeness, positive interactions and social time, ^a^random intercept model, ^b^random intercept and slopes model, ^c^model includes autocorrelation.

#### Effect of daily changes in sleep on social connectedness

**Figure 7:**
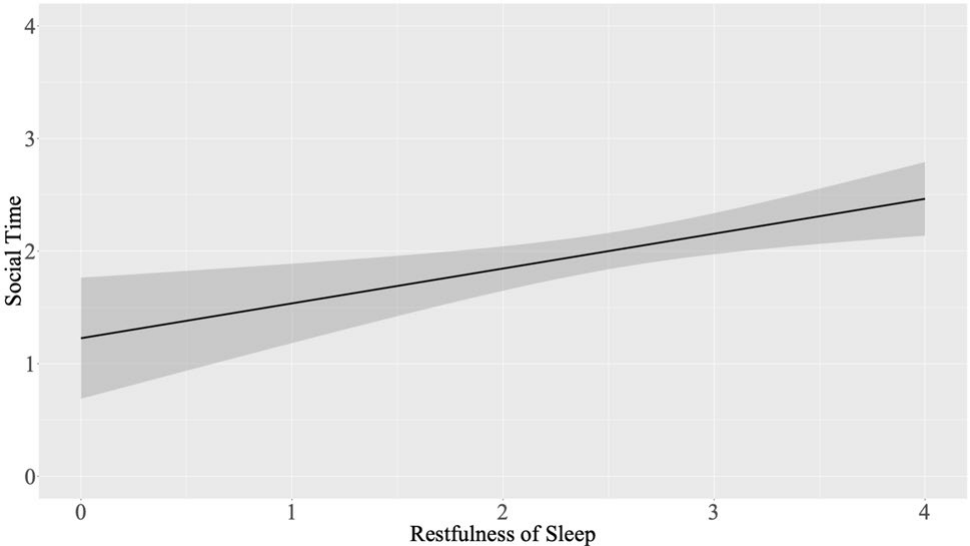
Predicting amount of time spent with family and friends from restfulness of sleep (state-level analyses t_1_). *Note.* X-Axis values for restfulness of sleep refer to the original (i.e., non-mean-centered) scores.

Greater restfulness of sleep statistically significantly predicted a greater amount of time spent with family and friends (B = 0.187, SE = 0.088, p = 0.034); Fig. 7 depicts the result. None of the remaining models were significant (all *p* > 0.092). See Table 9 for the results, and the supplementary material for the outcomes of the different versions of the models and the full final results table including the effect of stress (Tables S7 and S8).

**Table 9:** State-level analyses predicting social connectedness from sleep at t_1_ (n = 46).

Outcome	Predictors	B (SE)	95% CI	p
Social safeness	Diary			
	Restfulness of sleep^ac^	0.009 (0.048)	[− 0.086, 0.103]	0.852
	TST (hrs.)^ac^	− 0.003 (0.031)	[− 0.065, 0.059]	0.931
	SOL (min.)^ac^	0.005 (0.003)	[− 0.001, 0.011]	0.102
	WUI^ac^	− 0.022 (0.237)	[− 0.488, 0.445]	0.926
	Wearable			
	TST (hrs.)^a^	− 0.041 (0.042)	[− 0.123, 0.042]	0.329
	WUI^a^	− 0.052 (0.065)	[− 0.180, 0.076]	0.422
	Nocturnal HR^a^	0.002 (0.008)	[− 0.014, 0.019]	0.794
Social time	Diary			
	Restfulness of sleep^ac^	0.187 (0.088)	[0.014, 0.360]	0.034*
	TST (hrs.)^ac^	0.101 (0.060)	[− 0.017, 0.218]	0.093
	SOL (min)^ac^	0.007 (0.007)	[− 0.006, 0.020]	0.298
	WUI^ac^	0.428 (0.458)	[− 0.475, 1.331]	0.351
	Wearable			
	TST (hrs.)^a^	0.047 (0.072)	[− 0.096, 0.190]	0.519
	WUI^a^	0.033 (0.100)	[− 0.165, 0.231]	0.740
	Nocturnal HR^a^	− 0.003 (0.012)	[− 0.027, 0.022]	0.833
Positive interactions	Diary			
	Restfulness of sleep^a^	0.044 (0.043)	[− 0.041, 0.130]	0.309
	TST (hrs.)^a^	− 0.034 (0.030)	[− 0.093, 0.025]	0.257
	SOL (min.)^a^	0.003 (0.004)	[− 0.004, 0.010]	0.342
	WUI^a^	0.007 (0.230)	[− 0.446, 0.461]	0.975
	Wearable			
	TST (hrs.)^a^	0.016 (0.036)	[− 0.055, 0.087]	0.656
	WUI^a^	− 0.051 (0.052)	[− 0.155, 0.052]	0.330
	Nocturnal HR^a^	− 0.008 (0.007)	[− 0.021, 0.006]	0.262

*Note.* TST, total sleep time; SOL, sleep onset latency; WUI, wake-up index (no. awakenings/TST); HR, heart rate; Social time, amount of time spent with family/friends; positive interactions, number of daytime positive social interactions; B, regression coefficient for the fixed effect; SE, standard error; CI, confidence interval; * = p < 0.05, ** = p < 0.01, *** = p < 0.001, grand-mean-centered scores were used for self-reported restfulness of sleep, ^a^random intercept model, ^b^random intercept and slopes model, ^c^model includes autocorrelation.

## Discussion

The aim of the present study was to examine bi-directional associations among sleep and loneliness/social connectedness on state- and trait-level in a sample of stress-exposed individuals in order to shed light on the temporal dynamics and direction of this association. Importantly, we extend upon previous research by investigating day-to-day associations using both objective as well as self-reported sleep indices. Taken together, we found indications for a bi-directional association among sleep and loneliness/social connectedness.

### Loneliness/Social connectedness predicting sleep

When examining the effect of loneliness/social connectedness on sleep, we found that, on trait-level, greater loneliness at baseline was a statistically significant predictor of higher daytime dysfunction and poorer global sleep quality three months later. Investigating daily changes in social connectedness and sleep, state-level analyses at t_0_ revealed that a higher number of positive social interactions during the day statistically significantly predicted a shorter wearable-based TST, such that an increase from “one” to “many” positive interactions predicted almost a forty-minutes reduction in wearable-based TST. Similarly, state-level analyses at t_1_ pointed at a positive impact of social safeness on restfulness of sleep, such that higher levels of social safeness was found to predict greater restfulness of sleep. In addition, an increase in the number of positive social interactions from “one” to “many” statistically significantly predicted an 8-min decrease in self-reported SOL as well as a lower wearable-based WUI during the following night. Our findings are in line with previous longitudinal studies showing the effect of loneliness on global sleep^20^ as well as with results of other intensive longitudinal studies highlighting the impact of daily changes in social connectedness on sleep^30,32^. Although previous studies have pointed at a different impact of positive (e.g., social connectedness) and negative (e.g., loneliness) social constructs on health^53^, our findings on social connectedness and loneliness seem comparable and suggest an influence of both variables on sleep. It has long been argued, that as humans have to rely on others in order to survive, feeling lonely or less socially safe is similar to feelings of threat or vulnerability, which can cause hypervigilance to social threats in lonely individuals^26–28^. This hypervigilance can be seen as an increased disposition to arousal during wakefulness and sleep, thus resulting in poorer sleep. Our findings support these assumptions and demonstrate the role loneliness and social connections play in healthy sleep.

Although findings of the present study generally speak to a positive impact of social connectedness and negative impact of loneliness on sleep, our results regarding the specific influence of positive social interactions on sleep are inconclusive. While at t_1_ a greater number of positive social interactions was affiliated with better sleep (as indicated by a shorter self-reported SOL and a lower wearable-based WUI), at t_0_ a greater number of positive social interactions was found to predict a shorter self-reported TST, thus implying a rather negative impact. While, generally, the beneficial effects of positive interactions and relationships on sleep have been highlighted in the literature^22,23^, some intensive longitudinal studies have also documented a rather negative influence of daily social interactions on daily sleep. For example, Holding and colleagues^35^ found an extended duration of social activity (> five hours) to be related to a decrease in sleep time. Similarly, Sladek and Doane^32^ observed that within-person increases in daily social connection predicted longer SOL for those low on trait loneliness. Here, the authors argued that the counterintuitive negative association between (greater) social connection and (longer) SOL in individuals low on trait-level loneliness might be understandable when within-person increases in daily social connection of individuals low on trait-level loneliness (compared to their already high average level) are seen as a parameter for a more challenging day, leading to difficulties falling asleep. Thus, these findings, along with our own, highlight the fact that while positive social interactions have beneficial effects, they may also be demanding or challenging at times resulting in poorer sleep. Another potential explanation for our contradictory findings may also lie in the nature of the positive interaction variable, which might not have been sensitive enough to reliably capture variability in the number of positive social interactions. Indeed, the response scale of the item was rather coarse only ranging from “one” to “several” to “many” and did not allow for participants to indicate that they had zero positive interactions. Additionally, while at t_1_, the models indicated that the number of positive social interactions seemed to be somehow related to the control variable stress, this was not the case at t_0_ despite not change in trait-level stress between time points, further questioning the reliability of this variable.

Despite these theoretical explanations and methodological issues potentially explaining our contradicting findings, it is important to acknowledge the fact, that, in sum, findings did not seem to replicate across the two time points in the same sample. While trait-level findings and state-level results at t_1_ pointed at a similar direction of association, state-level analyses at t_0_ were contradictory. This highlights the fact that our results were not as robust and might potentially be also due to type I errors, which, in sum, significantly limits our ability to draw reliable conclusions based on the data.

### Sleep predicting loneliness/social connectedness

When examining the effect of sleep on loneliness/social connectedness, we found that, on trait-level, there was a trend towards higher daytime dysfunction at baseline predicting greater loneliness three months later. Investigating the influence of daily changes in sleep on social connectedness, a longer wearable-based TST was found to be a statistically significant predictor of less social safeness at t_0._ Additionally, greater restfulness of sleep statistically significantly predicted a lower number of positive social interactions at t_0_ and more time spent with family/friends at t_1_. Although generally research investigating the effect of sleep on loneliness/social connectedness is limited^20^, especially compared with evidence on the opposite direction, more and more research is emphasizing the key role of sleep for social processes^54,55^. Besides other intensive longitudinal studies suggesting an influence of sleep on loneliness/social connectedness^29,33–35^, there is also some evidence from experimental work pointing at a causal impact of sleep on social processes. For example, in their laboratory study, McMaking and colleagues^56^ observed levels of negative affect and reactivity to negative interactions with a friend to be increased in adolescents in a sleep deprivation vs. well-resting condition. Conversely, studies have documented beneficial socioemotional changes as a result of an increase and improvement in sleep (e.g., via cognitive behavioral therapy) [e.g.,^57,58^]. Several mechanisms have been suggested to underlie the causal impact of sleep on social processes with executive functioning and physiological arousal being among the most prominent^54,55^. In this regard, it has been argued that poor sleep may lead to impairments in executive functioning, such as reduced self-regulation and impairments in attention, as well as to increased physiological arousal which may in turn, lead to difficulties in social interactions and feelings of loneliness and a lack of connectedness in the long run. However, these mechanisms still remain relatively unexplored, and more research is needed to gain a more fine-grained understanding.

Although in trait-level analyses as well as state-level analyses at t_1,_ a rather positive impact of good sleep on loneliness/social connectedness was observed, which is in line with previous research as mentioned before, findings of state-level analyses at t_0_ pointed at a rather negative influence of good sleep on loneliness/social connectedness. Indeed, here, greater restfulness of sleep was found to be associated with a smaller number of positive social interactions and a longer wearable-based TST was found to predict less social safeness. Taking these findings together, results seemed to be rather conflicting across time points, speaking to a limited robustness of effects, which is important to consider when interpreting our findings. More research, particularly in larger samples, is needed in order to better understand the specific influence of sleep on interpersonal processes and feelings.

### Bi-directional association among sleep and loneliness

We identified one reciprocal association. On trait-level, we found that greater loneliness at baseline predicted higher daytime dysfunction three months later and that there was a trend towards higher daytime dysfunction at baseline, in turn, predicting greater loneliness three months later. These findings are in line with exising studies [e.g.,^33^]. Interestingly, previous research has shown that the higher reported daytime dysfunction of lonely individuals is independent of differences in sleep duration^59^ or in the activities of everyday life^60^. Instead, it has been suggested by Hawkley and colleagues^33^, that the higher daytime dysfunction in lonely individuals might rather be attributable to an increased number of nightly microawakenings, which has been identified in lonely individuals^26^. In our study, we did not find such an association among social safeness (i.e., lack of loneliness) and self-reported or wearable-based WUI on state-level, which could be due to the fact that our sample mainly consisted of relatively good sleepers. However, we did find a statistically significant association among a higher number of positive social interactions and a lower wearable-based WUI on state-level, which seems to be in line with previous findings. Taken together, our results suggest that the bi-directional interplay among loneliness and daytime dysfunctioning might resemble a downward spiral, in which both variables continue to negatively influence each other. More research is needed to understand the causal role microawakenings play, but also what exactly makes individuals vulnerable to microawakenings.

### Time scales of findings

In the current study, we investigated bi-directional associations between sleep and loneliness/social connectedness from both a trait and state perspective. While trait-level analyses investigated associations across a three-month period and looked at rather general patterns of loneliness and sleep, state-level analyses at t_0_ and t_1_ focused on day-to-day associations between daily changes in sleep and social connectedness. Results of trait-level associations pointed at a negative longer-term bi-directional association between loneliness and sleep, particularly with daytime dysfunction, highlighting the negative longer-term outcomes of both loneliness and poor sleep. In contrast, while state-level analyses generally indicated that social connectedness and sleep seemed to reciprocally influence each other from one day to the other, effects were much more fluctuating and inconsistent, particularly with regard to the valence of influence. Nevertheless, trait-level results suggested that these day-to-day effects, although being small and fluctuating, seem to negatively accumulate over time, leading to a vicious cycle of loneliness and sleep in the longer-run. Future research is needed to better understand the conditions, under which effects are more likely to negatively accumulate over time, as well as to identify the optimal timing for interventions (e.g., just-in-time adaptive interventions vs. time-unspecific treatment).

### Magnitude and reliability of statistically significant effects

As outlined above, in the present study, several associations between sleep and loneliness/social connectedness were statistically significant. Despite these interesting findings, overall, effects tended to be small and were only associated with minor changes in the respective dependent variable. Additionally, the number of analyses ran substantially increased likelihood of type-I errors, reducing the reliability of identified effects. Moreover, findings did not replicate across time points (e.g., differences in results between state-level analyses at t_0_ and t_1_), tended to be rather conflicting (e.g., positive vs. negative impact of good sleep on social connectedness observed at t_1_ vs. t_0_) and several were not in line with previous literature. On the other hand, as has also been noted by other researchers [e.g.,^61^], small effects should not be underestimated, as their cumulative impact—accumulated over time or aggregated across populations—may indeed have profound implications. In this regard, it is also important to consider that in the present study associations were investigated in a relatively healthy sample (i.e., effects may be larger in clinical populations) as well as that previous studies have already documented an accumulation of negative effects of both sleep and loneliness [e.g.,^62,63^]. Additionally, some of the current study’s results are routed in theory and have also been observed in previous studies. Nonetheless, we want to acknowledge these limitations here and strongly emphasize the fact, that they, in sum, substantially reduce our ability to draw any strong conclusions based on the data.

### Non-significant associations

A number of associations on both trait- and state-level were found to be non-significant. This is not in line with our hypotheses and differ from previous studies reporting robust associations among loneliness/social connectedness and sleep^18,19,22,23^. There are several possible explanations for this. First, our sample mainly consisted of relatively good sleepers, thus we may have not been able to detect statistically significant associations. Additionally, since previous studies have suggested that associations among affective variables and sleep might be particularly pronounced in the context of stress [e.g.,^24,25^], we investigated the loneliness-sleep associations in a sample of stress-exposed medical students during their first medical internship. However, average trait-level stress levels did not statistically significantly increase during the internship, which could be another possible explanation why we were not able to detect distinct associations as expected. Third, while the studies included in some of the previous meta-analyses on the sleep-loneliness/connectedness link focused rather on sleep problems (e.g., insomnia)^20,21^, most of our measures focused on sleep indices indicative of adaptive vs. maladaptive sleep, e.g., sleep duration.

### Practical implications

Results from the current study have practical implications. Our findings indicate that loneliness/social connectedness and sleep may be bi-directionally associated, given that we found associations in both directions. While, so far, research has predominantly focused on the impact of loneliness/social connectedness on sleep, a few other studies^29,33–35^ as well as the present investigation suggest that improvement of sleep may also help to reduce loneliness and increase social connectedness. Emotion regulation has been named as a potentially important factor contributing to the development of feelings of loneliness and a lack of social connectedness as a consequence of poor sleep^1,20^. Interventions teaching vulnerable individuals how to regulate their emotions in an adaptive way could potentially buffer the negative effects of poor sleep on feelings of loneliness and social connection. Moreover, sleep interventions could be an effective option to decrease loneliness and increase social connectedness in lonely individuals and patients, given the time-lagged effects of sleep and loneliness/social connectedness, which in turn could indirectly and further improve sleep quality^64,65^.

### Limitations

Findings of the present study have to be considered in light of several limitations. First, as our sample was a community sample, there was limited variance and frequency of sleep problems and feelings of loneliness/low social connectedness. Future studies are needed to uncover associations among sleep and loneliness/social connectedness in a clinical population. Second, we did not assess and control for the role of mood in state-level analyses; future research should investigate the influence of mood in day-to-day associations between sleep and social connectedness. Third, our items used to capture the number of positive social interactions as well as the amount of time spent with family/friends might not have been sensitive enough to capture the full range of social activities. Indeed, the item and response scale used for positive social interactions did not allow for participants to indicate that they had zero interactions. Additionally, the social time item focused on talking and listening to others, but did not capture other forms of social activity that do not involve any form of conversation (e.g., hugging); this may have biased our results and is important to consider when interpreting findings. Fourth, our sample consisted of medical students aged 22–27 years, thus limiting the generalizability of our findings to other age groups. More research is needed in order to shed light on the sleep-loneliness/social connectedness association across the life span.

## Conclusion

Taken together, the present study is an investigation of bi-directional trait- and state-level associations among sleep and loneliness/social connectedness in stress-exposed individuals. Our findings show a bi-directional relation among greater loneliness and higher daytime dysfunction on trait-level as well as point at several uni-directional associations on both trait- and state-level (i.e., observed in one but not the opposite direction). In sum, we found indications for both directions of the sleep-loneliness/social connectedness association. Findings of this study provide a more fine-grained understanding of the complex relationship between loneliness/social connectedness and sleep and thus also have implications for prevention and intervention science. Cognitive behavioral therapy has been found to be a promising approach for the treatment of both loneliness/low social connectedness as well as sleep problems [e.g.,^66,67^] and could be a key effective treatment option. Integrating the topic of social connection and loneliness in interventions targeting sleep problems, and vice verca could be beneficial and potentially buffer negative long-term effects.

### Supplementary Information

Supplementary Tables.

## Data Availability

All data and code are available on OSF (https://osf.io/e5awd/?view_only=b4122f52eb8846488454473c0be457d5).
