# Supplementary material for: Examining bi-directional links between loneliness, social connectedness and sleep from a trait and state perspective
Source: Sci Rep. 2024 Jul 27;14:17300. doi: 10.1038/s41598-024-68045-y (PMC11283477; doi:10.1038/s41598-024-68045-y)
Supplement: Supplementary file 1 — Supplementary Tables. [file 41598_2024_68045_MOESM1_ESM.pdf]

Supplementary Material

Examining bi-directional links between loneliness, social connectedness and sleep from a trait and state perspective

Christine Dworschak, Thomas Mäder, Charlotta Rühlmann, Andreas Maercker, & Birgit Kleim

Table S1

Outcomes of the different versions of the models and their comparisons (state-level analyses t_0; predicting sleep from social connectedness)

		**M1[a]**		**M2[b]**		**M3[c]**		**Model Comparison**		
**Outcome**	**Predictors**	**B (SE)**	**p**	**B (SE)**	**p**	**B (SE)**	**p**	**M1 vs. M2**	**M1 vs. M3**	**M2 vs. M3**
**Diary**										
Restfulness of sleep	Social safeness	0.150 (0.078)	.056	0.157 (0.081)	.056	0.148 (0.077)	.058	$\chi^2(2)$=0.329, p=.848	$\chi^2(1)$=0.098, p=.754	
	Social time	0.042 (0.043)	.327	0.056 (0.048)	.242	0.053 (0.041)	.197	$\chi^2(2)$=3.797, p=.150	$\chi^2(1)$=2.946, p=.086	
	Positive interactions	0.123 (0.084)	.144	0.104 (0.096)	.282	0.126 (0.083)	.131	$\chi^2(2)$=4.133, p=.127	$\chi^2(1)$=0.255, p=.614	
TST (hrs.)	Social safeness	0.137 (0.119)	.252	0.115 (0.117)	.326	0.167 (0.122)	.172	$\chi^2(2)$=1.456, p=.483	$\chi^2(1)$=0.918, p=.338	
	Social time	0.084 (0.064)	.195	0.087 (0.066)	.191	0.079 (0.063)	.215	$\chi^2(2)$=0.144, p=.931	$\chi^2(1)$=0.292, p=.589	
	Positive interactions	0.052 (0.126)	.679	0.050 (0.128)	.696	0.059 (0.126)	.643	$\chi^2(2)$=0.284, p=.868	$\chi^2(1)$=0.472, p=.492	
SOL (min.)	Social safeness	-0.752 (1.705)	.660	-2.351 (3.314)	.479	-2.432 (3.338)	.467	$\chi^2(2)$=19.511, p<.001***		$\chi^2(1)$=4.443, p=.035*
	Social time	-0.887 (0.967)	.360	-1.256 (1.055)	.236	-0.785 (0.90)	.384	$\chi^2(2)$=2.361, p=.307	$\chi^2(1)$=4.686, p=.030*	
	Positive interactions	-3.188 (1.767)	.073	-2.475 (2.519)	.327	-3.370 (1.744)	.055	$\chi^2(2)$=14.600, p<0.001***		$\chi^2(1)$=0.388, p=.533
WUI	Social safeness	0.009 (0.025)	.713	[d]		0.010 (0.025)	.705		$\chi^2(1)$=0.011, p=.915	
	Social time	0.006 (0.015)	.701	0.002 (0.016)	.910	0.006 (0.014)	.652	$\chi^2(2)$=1.028, p=.598	$\chi^2(1)$=0.841, p=.359	
	Positive interactions	-0.063 (0.028)	.025*	-0.044 (0.035)	.208	-0.045 (0.035)	.206	$\chi^2(2)$=13.432, p=.001**		$\chi^2(1)$=0.345, p=.557

**Wearable**										
TST (hrs.)	Social safeness	-0.129 (0.155)	.405	-0.178 (0.178)	.319	-0.185 (0.162)	.256	$\chi^2(2)=7.508$, p=.023*		$\chi^2(1)=3.963$, p=.047*
	Social time	0.002 (0.089)	.980	-0.008 (0.091)	.934	0.008 (0.084)	.925	$\chi^2(2)=1.524$, p=.467	$\chi^2(1)=1.744$, p=.187	
	Positive interactions	-0.336 (0.167)	.046*	-0.336 (0.167)	.046*	-0.380 (0.160)	.019*	$\chi^2(2)=0.001$, p=1.000	$\chi^2(1)=4.692$, p=.030*	
WUI	Social safeness	0.150 (0.111)	.179	0.185 (0.115)	.108	0.089 (0.082)	.281	$\chi^2(2)=2.224$, p=.329	$\chi^2(1)=5.277$, p=.022*	
	Social time	0.023 (0.058)	.686	0.007 (0.062)	.915	0.024 (0.057)	.677	$\chi^2(2)=0.881$, p=.644	$\chi^2(1)=0.272$, p=.602	
	Positive interactions	-0.139 (0.119)	.245	-0.142 (0.121)	.243	-0.117 (0.097)	.229	$\chi^2(2)=0.036$, p=.982	$\chi^2(1)=5.590$, p=.018*	

Note. TST = Total sleep time, SOL = Sleep onset latency, WUI = Wake-up index (no. awakenings / TST), HR = Heart rate, Social time = Amount of time spent with family/friends, Positive interactions = Number of daytime positive social interactions, B = regression coefficient for the fixed effect, SE = Standard error, * = p < .05, ** = p < .01, *** = p < .001, grand-mean-centered scores were used for social safeness, positive interactions and social time; [a]random intercept model; [b]random intercept and slopes model; [c]model including autocorrelation (either random intercept or random intercept and slopes model, depending on the results of the comparison of Model 1 vs. 2); [d]model did not converge; see data analysis section of the manuscript for a detailed description of the process.

Table S2

State-level analyses predicting sleep from social connectedness at t_0 (n=48) (full table including the effect of stress)

Outcome	Predictors	B (SE)	95% CI	p
**Diary**				
Restfulness of sleep	Social safeness	0.126 (0.078)	[-0.028, 0.279]	.109
	Stress	-0.004 (0.002)	[-0.008, -0.000]	.039*
	Social Time	0.027 (0.044)	[-0.059, 0.113]	.536
	Stress	-0.004 (0.002)	[-0.008, 0.000]	.083
	Positive interactions	0.131 (0.084)	[-0.034, 0.296]	.120
	Stress	-0.005 (0.002)	[-0.009, -0.001]	.012*
TST (hrs.)	Social safeness	0.154 (0.123)	[-0.090, 0.397]	.215
	Stress	-0.000 (0.003)	[-0.006, 0.005]	.877
	Social time	0.080 (0.066)	[-0.050, 0.210]	.225
	Stress	0.000 (0.003)	[-0.006, 0.007]	.901
	Positive interactions	0.055 (0.127)	[-0.196, 0.305]	.668
	Stress	-0.001 (0.003)	[-0.007, 0.004]	.621
SOL (min.)	Social safeness	-3.369 (3.415)	[-10.096, 3.358]	.325
	Stress	-0.076 (0.039)	[-0.153, 0.002]	.055
	Social time	-1.171 (0.924)	[-2.995, 0.652]	.207
	Stress	-0.108 (0.044)	[-0.195, -0.021]	.015*
	Positive interactions	-2.745 (1.793)	[-6.277, 0.788]	.127
	Stress	-0.065 (0.041)	[-0.144, 0.015]	.113
WUI	Social safeness	0.017 (0.026)	[-0.034, 0.068]	.514
	Stress	0.001 (0.001)	[-0.000, 0.002]	.175
	Social time	0.006 (0.015)	[-0.023, 0.036]	.677
	Stress	0.001 (0.001)	[-0.001, 0.002]	.375
	Positive interactions	-0.039 (0.036)	[-0.110, 0.031]	.273
	Stress	0.001 (0.001)	[-0.001, 0.002]	.367
**Wearable**				
TST (hrs.)	Social safeness	-0.269 (0.169)	[-0.602, 0.065]	.114
	Stress	-0.010 (0.004)	[-0.017, -0.002]	.011*
	Social time	-0.048 (0.090)	[-0.226, 0.130]	.596
	Stress	-0.009 (0.004)	[-0.018, -0.000]	.047*
	Positive interactions	-0.327 (0.164)	[-0.651, -0.004]	.048*
	Stress	-0.008 (0.004)	[-0.016, -0.001]	.036*
WUI	Social safeness	0.160 (0.111)	[-0.059, 0.379]	.152
	Stress	0.002 (0.003)	[-0.004, 0.008]	.457
	Social time	0.036 (0.060)	[-0.083, 0.156]	.550
	Stress	0.001 (0.003)	[-0.005, 0.007]	.698
	Positive interactions	-0.173 (0.118)	[-0.407, 0.060]	.145
	Stress	0.001 (0.003)	[-0.004, 0.007]	.686

Note. TST = Total sleep time, SOL = Sleep onset latency, WUI = Wake-up index (no. awakenings / TST), HR = Heart rate, Social time = Amount of time spent with family/friends, Positive interactions = Number of daytime positive social interactions, B = regression coefficient for the fixed effect, SE = Standard error, CI = Confidence interval, * = p < .05, ** = p < .01, *** = p < .001, grand-mean-centered scores were used for social safeness, positive interactions, social time and stress.

Table S3

Outcomes of the different versions of the models and their comparisons (state-level analyses t_0; predicting social connectedness from sleep)

		M1[a]		M2[b]		M3[c]		Model Comparison		
**Outcome**	**Predictors**	**B (SE)**	**p**	**B (SE)**	**p**	**B (SE)**	**p**	**M1 vs. M2**	**M1 vs. M3**	**M2 vs. M3**
Social safeness	**Diary**									
	Restfulness of sleep	-0.000 (0.040)	.999	-0.005 (0.041)	.897	-0.007 (0.037)	.852	χ^2(2)=3.040, p=.219	χ^2(1)=9.942, p=.002**	
	TST (hrs.)	-0.020 (0.028)	.483	-0.036 (0.028)	.209	-0.010 (0.024)	.676	χ^2(2)=0.655, p=.721	χ^2(1)=10.890, p=.001**	
	SOL (min.)	0.001 (0.002)	.476	0.001 (0.002)	.663	0.001 (0.002)	.405	χ^2(2)=0.159, p=.924	χ^2(1)=9.985, p=.002**	
	WUI	-0.100 (0.156)	.523	-0.157 (0.167)	.348	-0.026 (0.154)	.866	χ^2(2)=0.198, p=.906	χ^2(1)=2.817, p=.093	
	**Wearable**									
	TST (hrs.)	-0.070 (0.032)	.030*	[d]		-0.072 (0.031)	.023*		χ^2(1)=1.334, p=.248	
	WUI	-0.056 (0.048)	.242	-0.068 (0.049)	.170	-0.052 (0.047)	.268	χ^2(2)=0.483, p=.785	χ^2(1)=0.745, p=.388	
Social Time	**Diary**									
	Restfulness of sleep	-0.012 (0.098)	.902	[d]		-0.015 (0.098)	.875		χ^2(1)= 0.025, p=.874	
	TST (hrs.)	0.039 (0.065)	.551	[d]		0.039 (0.065)	.543		χ^2(1)=0.027, p=.869	
	SOL (min.)	0.006 (0.004)	.182	0.006 (0.004)	.182	0.006 (0.004)	.161	χ^2(2)=0.001, p=.999	χ^2(1)=0.050, p=.823	
	WUI	0.163 (0.372)	.663	0.157 (0.389)	.688	0.154 (0.372)	.680	χ^2(2)=0.004, p=.998	χ^2(1)=0.101, p=.751	
	**Wearable**									
	TST (hrs.)	-0.027 (0.073)	.712	-0.027 (0.073)	.712	-0.025 (0.073)	.732	χ^2(2)=0.000, p=1.000	χ^2(1)=0.045, p=.833	
	WUI	-0.021 (0.112)	.852	-0.021 (0.112)	.852	-0.020 (0.111)	.860	χ^2(2)=0.001, p=.999	χ^2(1)=0.054, p=.816	
Positive Interactions	**Diary**									
	Restfulness of sleep	-0.123 (0.041)	.003**	-0.117 (0.045)	.010*	-0.124 (0.041)	.003**	χ^2(2)=0.817, p=.665	χ^2(1)=0.075, p=.785	

TST (hrs.)	-0.031 (0.029)	.285	-0.032 (0.031)	.308	-0.031 (0.029)	.285	$\chi^2(2)=0.384$, p=.825	$\chi^2(1)=0.001$, p=.981
SOL (min.)	0.000 (0.002)	.802	0.001 (0.003)	.593	0.000 (0.002)	.802	$\chi^2(2)=0.884$, p=.643	$\chi^2(1)=0.000$, p=.992
WUI	0.279 (0.163)	.088	0.442 (0.201)	.029*	0.336 (0.159)	.036*	$\chi^2(2)=4.923$, p=.085	$\chi^2(1)=2.787$, p=.095
**Wearable**								
TST (hrs.)	-0.031 (0.032)	.335	-0.041 (0.037)	.277	-0.032 (0.032)	.314	$\chi^2(2)=0.812$, p=.666	$\chi^2(1)=1.790$, p=.181
WUI	-0.000 (0.048)	.992	-0.000 (0.048)	.993	0.013 (0.047)	.782	$\chi^2(2)=0.005$, p=.997	$\chi^2(1)=1.738$, p=.187

Note. TST = Total sleep time, SOL = Sleep onset latency, WUI = Wake-up index (no. awakenings / TST), HR = Heart rate, Social time = Amount of time spent with family/friends, Positive interactions = Number of daytime positive social interactions, B = Regression coefficient for the fixed effect, SE = Standard error, CI = Confidence interval, * = p < .05, ** = p < .01, *** = p < .001, grand-mean-centered scores were used for self-reported restfulness of sleep; [a]random intercept model; [b]random intercept and slopes model; [c]model including autocorrelation (either random intercept or random intercept and slopes model, depending on the results of the comparison of Model 1 vs. 2); [d]model did not converge; see data analysis section of the manuscript for a detailed description of the process.

Table S4

State-level analyses predicting social connectedness from sleep at t_0 (n=48) (full table including the effect of stress)

Outcome	Predictors	B (SE)	95% CI	p
Social safeness	**Diary**			
	Restfulness of sleep	-0.015 (0.040)	[-0.094, 0.063]	.700
	Stress	-0.004 (0.001)	[-0.007, -0.002]	.001**
	TST (hrs.)	-0.032 (0.028)	[-0.086, 0.023]	.251
	Stress	-0.004 (0.001)	[-0.007, -0.002]	.001**
	SOL (min.)	0.001 (0.002)	[-0.002, 0.005]	.460
	Stress	-0.004 (0.001)	[-0.007, -0.002]	.001**
	WUI	-0.058 (0.154)	[-0.361, 0.245]	.706
	Stress	-0.005 (0.001)	[-0.007, -0.002]	.002**
	**Wearable**			
	TST (hrs.)	-0.068 (0.031)	[-0.130, -0.006]	.031*
	Stress	-0.007 (0.002)	[-0.010, -0.003]	<.001***
	WUI	-0.045 (0.048)	[-0.140, 0.049]	.345
	Stress	-0.007 (0.002)	[-0.010, -0.003]	<.001***
Social time	**Diary**			
	Restfulness of sleep	-0.050 (0.106)	[-0.258, 0.159]	.640
	Stress	-0.003 (0.003)	[-0.009, 0.004]	.407
	TST (hrs.)	0.028 (0.068)	[-0.107, 0.164]	.680
	Stress	-0.002 (0.003)	[-0.008, 0.005]	.554
	SOL (min)	0.007 (0.004)	[-0.001, 0.016]	.105
	Stress	-0.002 (0.003)	[-0.009, 0.004]	.451
	WUI	0.194 (0.389)	[-0.577, 0.966]	.619
	Stress	-0.001 (0.004)	[-0.008, 0.006]	.689
	**Wearable**			
	TST (hrs.)	-0.021 (0.077)	[-0.175, 0.132]	.783
	Stress	-0.006 (0.004)	[-0.014, 0.003]	.188
	WUI	0.034 (0.118)	[-0.200, 0.268]	.773
	Stress	-0.006 (0.004)	[-0.014, 0.003]	.184
Positive Interactions	**Diary**			
	Restfulness of sleep	-0.117 (0.042)	[-0.201, -0.034]	.006**
	Stress	0.001 (0.001)	[-0.002, 0.004]	.451
	TST (hrs.)	-0.027 (0.029)	[-0.085, 0.031]	.364
	Stress	0.001 (0.001)	[-0.001, 0.004]	.279
	SOL (min.)	0.001 (0.002)	[-0.003, 0.004]	.787
	Stress	0.002 (0.001)	[-0.001, 0.004]	.212
	WUI	0.281 (0.166)	[-0.046, 0.608]	.092
	Stress	0.001 (0.001)	[-0.002, 0.004]	.345
	**Wearable**			
	TST (hrs.)	-0.030 (0.033)	[-0.095, 0.035]	.358
	Stress	0.001 (0.002)	[-0.003, 0.004]	.616
	WUI	-0.015 (0.049)	[-0.112, 0.082]	.755
	Stress	0.001 (0.002)	[-0.003, 0.005]	.572

Note. TST = Total sleep time, SOL = Sleep onset latency, WUI = Wake-up index (no. awakenings / TST), HR = Heart rate, Social time = Amount of time spent with family/friends, Positive interactions = Number of daytime positive social interactions, B = Regression coefficient for the fixed effect, SE = Standard error, CI = Confidence interval, * = p < .05, ** = p < .01, *** = p < .001, grand-mean-centered scores were used for self-reported restfulness of sleep and stress.

Table S5

Outcomes of the different versions of the models and their comparisons (state-level analyses t_1; predicting sleep from social connectedness)

		M1[a]		M2[b]		M3[c]		Model Comparison		
**Outcome**	**Predictors**	**B (SE)**	**p**	**B (SE)**	**p**	**B (SE)**	**p**	**M1 vs. M2**	**M1 vs. M3**	**M2 vs. M3**
**Diary**										
Restfulness of sleep	Social safeness	0.120 (0.057)	.036*	0.107 (0.063)	.089	0.106 (0.053)	.046*	$\chi^2(2)$=1.190, p=.552	$\chi^2(1)$=0.460, p=.498	
	Social time	0.091 (0.036)	.013*	0.091 (0.036)	.013*	0.081 (0.033)	.016*	$\chi^2(2)$=0.002, p=.999	$\chi^2(1)$=0.841, p=.359	
	Positive interactions	0.019 (0.075)	.800	0.003 (0.095)	.972	0.033 (0.070)	.633	$\chi^2(2)$=3.259, p=.196	$\chi^2(1)$=1.084, p=.298	
TST (hrs.)	Social safeness	-0.005 (0.094)	.958	-0.005 (0.094)	.958	0.008 (0.076)	.920	$\chi^2(2)$=0.000, p=1.00	$\chi^2(1)$=4.488, p=.034*	
	Social time	0.013 (0.058)	.819	0.024 (0.061)	.689	-0.026 (0.049)	.601	$\chi^2(2)$=1.982, p=.371	$\chi^2(1)$=4.846, p=.028*	
	Positive interactions	-0.052 (0.124)	.674	-0.053 (0.124)	.672	-0.068 (0.107)	.526	$\chi^2(2)$=0.003, p=.999	$\chi^2(1)$=4.835, p=.028*	
SOL (min.)	Social safeness	-1.271 (0.765)	.098	-1.068 (0.750)	.156	-1.213 (0.771)	.117	$\chi^2(2)$=1.490, p=.475	$\chi^2(1)$=0.944, p=.331	
	Social time	-0.752 (0.402)	.063	-0.752 (0.402)	.063	-0.836 (0.414)	.044*	$\chi^2(2)$=0.003, p=.999	$\chi^2(1)$=1.808, p=.179	
	Positive interactions	-3.837 (0.923)	<.0001***	-4.286 (1.422)	.003**	-4.312 (1.442)	.003**	$\chi^2(2)$=7.217, p=.027*		$\chi^2(1)$=0.097, p=.755
WUI	Social safeness	0.020 (0.016)	.195	[d]		0.013 (0.014)	.347		$\chi^2(1)$=4.029, p=.045*	
	Social time	-0.004 (0.009)	.644	-0.004 (0.009)	.644	-0.003 (0.008)	.652	$\chi^2(2)$=0.001, p=.999	$\chi^2(1)$=4.891, p=.027*	
	Positive interactions	-0.001 (0.020)	.979	0.008 (0.023)	.714	0.002 (0.018)	.924	$\chi^2(2)$=5.715, p=.057	$\chi^2(1)$=4.933, p=.026*	
**Wearable**										
TST (hrs.)	Social safeness	-0.053 (0.135)	.695	-0.053 (0.135)	.696	-0.048 (0.132)	.716	$\chi^2(2)$=0.001, p=.999	$\chi^2(1)$=0.161, p=.688	
	Social time	0.138 (0.080)	.085	0.139 (0.080)	.085	0.141 (0.080)	.080	$\chi^2(2)$=0.006, p=.997	$\chi^2(1)$=0.008, p=.930	

	Positive interactions	0.013 (0.177)	.940	0.043 (0.184)	.814	-0.004 (0.173)	.979	$\chi^2(2)$=1.288, p=.525	$\chi^2(1)$=0.190, p=.663
WUI	Social safeness	-0.097 (0.073)	.183	-0.069 (0.116)	.553	-0.092 (0.062)	.142	$\chi^2(2)$=5.865, p=.053	$\chi^2(1)$=5.743, p=.017*
	Social time	-0.090 (0.047)	.055	[d]		-0.077 (0.041)	.067		$\chi^2(1)$=5.083, p=.024*
	Positive interactions	-0.245 (0.101)	.017*	-0.215 (0.122)	.081	-0.224 (0.091)	.015*	$\chi^2(2)$=2.315, p=.314	$\chi^2(1)$=5.731, p=.017*
Nocturnal HR	Social safeness	0.358 (0.582)	.539	0.358 (0.582)	.539	0.164 (0.479)	.733	$\chi^2(2)$=0.002, p=.999	$\chi^2(1)$=8.228, p=.004**
	Social time	0.287 (0.374)	.444	0.287 (0.374)	.445	0.210 (0.322)	.514	$\chi^2(2)$=0.001, p=1.000	$\chi^2(1)$=8.421, p=.004**
	Positive interactions	0.386 (0.796)	.628	0.385 (0.796)	.629	-0.022 (0.678)	.975	$\chi^2(2)$=0.003, p=.999	$\chi^2(1)$=8.332, p=.004**

Note. TST = Total sleep time, SOL = Sleep onset latency, WUI = Wake-up index (no. awakenings / TST), HR = Heart rate, Social time = Amount of time spent with family/friends, Positive interactions = Number of daytime positive social interactions, B = regression coefficient for the fixed effect, SE = Standard error, * = p < .05, ** = p < .01, *** = p < .001, grand-mean-centered scores were used for social safeness, positive interactions and social time; [a]random intercept model; [b]random intercept and slopes model; [c]model including autocorrelation (either random intercept or random intercept and slopes model, depending on the results of the comparison of Model 1 vs. 2); [d]model did not converge; see data analysis section of the manuscript for a detailed description of the process.

Table S6

State-level analyses predicting sleep from social connectedness at t_1 (n=46) (full table including the effect of stress)

Outcome	Predictors	B (SE)	95% CI	p
**Diary**				
Restfulness of sleep	Social safeness	0.128 (0.059)	[0.012, 0.244]	.031*
	Stress	-0.005 (0.002)	[-0.009, -0.001]	.019*
	Social Time	0.045 (0.041)	[-0.036, 0.127]	.273
	Stress	-0.005 (0.002)	[-0.009, -0.001]	.025*
	Positive interactions	0.047 (0.079)	[-0.109, 0.203]	.553
	Stress	-0.005 (0.002)	[-0.009, -0.001]	.013*
TST (hrs.)	Social safeness	-0.026 (0.096)	[-0.216, 0.164]	.789
	Stress	-0.006 (0.003)	[-0.013, 0.000]	.054
	Social time	-0.125 (0.064)	[-0.250, 0.000]	.051
	Stress	-0.007 (0.003)	[-0.013, -0.000]	.041*
	Positive interactions	-0.026 (0.130)	[-0.282, 0.231]	.845
	Stress	-0.006 (0.003)	[-0.013, 0.000]	.067
SOL (min.)	Social safeness	-1.252 (0.784)	[-2.797, 0.293]	.112
	Stress	0.025 (0.024)	[-0.023, 0.073]	.313
	Social time	-0.758 (0.488)	[-1.720, 0.204]	.122
	Stress	0.020 (0.025)	[-0.029, 0.069]	.422
	Positive interactions	-4.324 (1.379)	[-7.042, -1.607]	.002**
	Stress	0.041 (0.024)	[-0.005, 0.088]	.082
WUI	Social safeness	0.023 (0.016)	[-0.008, 0.055]	.146
	Stress	0.000 (0.001)	[-0.001, 0.001]	.394
	Social time	-0.003 (0.010)	[-0.022, 0.016]	.756
	Stress	0.000 (0.000)	[-0.001, 0.001]	.668
	Positive interactions	0.002 (0.020)	[-0.037, 0.041]	.924
	Stress	0.000 (0.000)	[-0.001, 0.001]	.648
**Wearable**				
TST (hrs.)	Social safeness	-0.154 (0.136)	[-0.424, 0.116]	.262
	Stress	-0.010 (0.004)	[-0.019, -0.002]	.021*
	Social time	0.039 (0.086)	[-0.132, 0.209]	.656
	Stress	-0.009 (0.004)	[-0.018, -0.001]	.036*
	Positive interactions	0.097 (0.179)	[-0.256, 0.451]	.587
	Stress	-0.010 (0.004)	[-0.019, -0.001]	.029*
WUI	Social safeness	-0.083 (0.066)	[-0.213, 0.047]	.210
	Stress	-0.000 (0.003)	[-0.005, 0.005]	.928
	Social time	-0.091 (0.049)	[-0.188, 0.006]	.066
	Stress	-0.000 (0.003)	[-0.005, 0.005]	.986
	Positive interactions	-0.280 (0.100)	[-0.478, -0.081]	.006**
	Stress	0.001 (0.002)	[-0.004, 0.006]	.579
Nocturnal HR	Social safeness	0.253 (0.573)	[-0.881, 1.388]	.659
	Stress	0.011 (0.020)	[-0.029, 0.052]	.580
	Social time	0.238 (0.411)	[-0.576, 1.051]	.564
	Stress	0.012 (0.020)	[-0.028, 0.053]	.546
	Positive interactions	-0.025 (0.801)	[-1.612, 1.562]	.976
	Stress	0.009 (0.021)	[-0.031, 0.050]	.648

Note. TST = Total sleep time, SOL = Sleep onset latency, WUI = Wake-up index (no. awakenings / TST), HR = Heart rate, Social time = Amount of time spent with family/friends, Positive interactions = Number of daytime positive social interactions, B = regression coefficient for the fixed effect, SE = Standard error, CI = Confidence interval, * = p < .05, ** = p < .01, *** = p < .001, grand-mean-centered scores were used for social safeness, positive interactions, social time and stress.

Table S7

Outcomes of the different versions of the models and their comparisons (state-level analyses t_1; predicting social connectedness from sleep)

		M1[a]		M2[b]		M3[c]		Model Comparison		
**Outcome**	**Predictors**	**B (SE)**	**p**	**B (SE)**	**p**	**B (SE)**	**p**	**M1 vs. M2**	**M1 vs. M3**	**M2 vs. M3**
Social safeness	**Diary**									
	Restfulness of sleep	0.055 (0.053)	.303	0.042 (0.064)	.506	0.005 (0.045)	.913	$\chi^2(2)=3.695$, p=.158	$\chi^2(1)=18.142$, p<.0001***	
	TST (hrs.)	-0.023 (0.036)	.511	-0.021 (0.036)	.560	0.002 (0.029)	.949	$\chi^2(2)=1.285$, p=.526	$\chi^2(1)=17.084$, p<.0001***	
	SOL (min.)	0.004 (0.004)	.365	0.004 (0.004)	.365	0.005 (0.003)	.094	$\chi^2(2)=0.005$, p=.998	$\chi^2(1)=20.674$, p<.0001***	
	WUI	-0.150 (0.282)	.595	-0.145 (0.283)	.610	0.014 (0.230)	.950	$\chi^2(2)=0.005$, p=.997	$\chi^2(1)=10.891$, p=.001**	
	**Wearable**									
	TST (hrs.)	-0.016 (0.041)	.709	-0.016 (0.041)	.709	-0.015 (0.041)	.712	$\chi^2(2)=0.007$, p=.997	$\chi^2(1)=0.008$, p=.928	
	WUI	-0.054 (0.065)	.403	-0.072 (0.068)	.292	-0.054 (0.065)	.404	$\chi^2(2)=1.045$, p=.593	$\chi^2(1)=0.046$, p=.831	
	Nocturnal HR	0.006 (0.008)	.428	0.006 (0.008)	.428	0.006 (0.008)	.419	$\chi^2(2)=0.004$, p=.998	$\chi^2(1)=0.181$, p=.671	
Social Time	**Diary**									
	Restfulness of sleep	0.284 (0.096)	.004**	0.299 (0.111)	.008**	0.276 (0.094)	.004**	$\chi^2(2)=1.137$, p=.566	$\chi^2(1)=9.763$, p=.002**	
	TST (hrs.)	0.139 (0.064)	.031*	0.148 (0.072)	.040*	0.131 (0.062)	.036*	$\chi^2(2)=0.967$, p=.617	$\chi^2(1)=8.189$, p=.004**	
	SOL (min.)	0.004 (0.007)	.565	0.003 (0.007)	.650	0.004 (0.008)	.602	$\chi^2(2)=0.010$, p=.995	$\chi^2(1)=9.830$, p=.002**	
	WUI	0.331 (0.485)	.496	0.235 (0.593)	.692	0.449 (0.486)	.357	$\chi^2(2)=1.272$, p=.529	$\chi^2(1)=8.323$, p=.004**	
	**Wearable**									
	TST (hrs.)	0.088 (0.077)	.253	0.114 (0.106)	.282	0.082 (0.077)	.288	$\chi^2(2)=5.272$, p=.072	$\chi^2(1)=1.397$, p=.237	
	WUI	0.000 (0.099)	.996	0.002 (0.107)	.982	-0.009 (0.105)	.935	$\chi^2(2)=0.394$, p=.821	$\chi^2(1)=1.656$, p=.198	
	Nocturnal HR	0.007 (0.011)	.523	0.007 (0.011)	.510	0.008 (0.012)	.534	$\chi^2(2)=0.007$, p=.996	$\chi^2(1)=1.765$, p=.184	

Positive Interactions	**Diary**								
	Restfulness of sleep	0.051 (0.043)	.233	0.039 (0.050)	.435	0.051 (0.043)	.237	$\chi^2(2)=3.738$, p=.154	$\chi^2(1)=0.007$, p=.933
	TST (hrs.)	-0.032 (0.029)	.275	-0.031 (0.029)	.288	-0.032 (0.029)	.278	$\chi^2(2)=0.027$, p=.986	$\chi^2(1)=0.008$, p=.930
	SOL (min.)	0.003 (0.004)	.333	0.003 (0.004)	.339	0.004 (0.004)	.329	$\chi^2(2)=0.003$, p=.999	$\chi^2(1)=0.012$, p=.913
	WUI	-0.122 (0.229)	.595	-0.122 (0.229)	.596	-0.134 (0.230)	.559	$\chi^2(2)=0.002$, p=.999	$\chi^2(1)=0.084$, p=.772
	**Wearable**								
	TST (hrs.)	0.009 (0.036)	.798	0.018 (0.039)	.652	0.008 (0.036)	.831	$\chi^2(2)=1.017$, p=.601	$\chi^2(1)=1.035$, p=.309
	WUI	-0.057 (0.053)	.288	[d]		-0.069 (0.052)	.182		$\chi^2(1)=1.558$, p=.212
	Nocturnal HR	-0.002 (0.006)	.783	-0.003 (0.007)	.671	-0.002 (0.006)	.802	$\chi^2(2)=0.683$, p=.711	$\chi^2(1)=1.014$, p=.314

Note. TST = Total sleep time, SOL = Sleep onset latency, WUI = Wake-up index (no. awakenings / TST), HR = Heart rate, Social time = Amount of time spent with family/friends, Positive interactions = Number of daytime positive social interactions, B = Regression coefficient for the fixed effect, SE = Standard error, CI = Confidence interval, * = $p < .05$, ** = $p < .01$, *** = $p < .001$, grand-mean-centered scores were used for self-reported restfulness of sleep; [a]random intercept model; [b]random intercept and slopes model; [c]model including autocorrelation (either random intercept or random intercept and slopes model, depending on the results of the comparison of Model 1 vs. 2); [d]model did not converge; see data analysis section of the manuscript for a detailed description of the process.

Table S8

State-level analyses predicting social connectedness from sleep at t_1 (n=46) (full table including the effect of stress)

Outcome	Predictors	B (SE)	95% CI	p
Social safeness	**Diary**			
	Restfulness of sleep	0.009 (0.048)	[-0.086, 0.103]	.852
	Stress	-0.001 (0.001)	[-0.003, 0.002]	.708
	TST (hrs.)	-0.003 (0.031)	[-0.065, 0.059]	.931
	Stress	-0.001 (0.001)	[-0.004, 0.002]	.591
	SOL (min.)	0.005 (0.003)	[-0.001, 0.011]	.102
	Stress	-0.001 (0.001)	[-0.003, 0.002]	.594
	WUI	-0.022 (0.237)	[-0.488, 0.445]	.926
	Stress	-0.001 (0.002)	[-0.004, 0.002]	.497
	**Wearable**			
	TST (hrs.)	-0.041 (0.042)	[-0.123, 0.042]	.329
	Stress	-0.005 (0.002)	[-0.009, -0.000]	.043*
	WUI	-0.052 (0.065)	[-0.180, 0.076]	.422
	Stress	-0.004 (0.002)	[-0.009, 0.000]	.055
	Nocturnal HR	0.002 (0.008)	[-0.014, 0.019]	.794
	Stress	-0.004 (0.002)	[-0.009, 0.000]	.063
Social time	**Diary**			
	Restfulness of sleep	0.187 (0.088)	[0.014, 0.360]	.034*
	Stress	-0.006 (0.003)	[-0.012, 0.000]	.057
	TST (hrs.)	0.101 (0.060)	[-0.017, 0.218]	.093
	Stress	-0.006 (0.003)	[-0.012, 0.001]	.076
	SOL (min)	0.007 (0.007)	[-0.006, 0.020]	.298
	Stress	-0.006 (0.003)	[-0.012, -0.000]	.040*
	WUI	0.428 (0.458)	[-0.475, 1.331]	.351
	Stress	-0.006 (0.003)	[-0.012, 0.000]	.051
	**Wearable**			
	TST (hrs.)	0.047 (0.072)	[-0.096, 0.190]	.519
	Stress	-0.003 (0.004)	[-0.010, 0.005]	.512
	WUI	0.033 (0.100)	[-0.165, 0.231]	.740
	Stress	-0.003 (0.004)	[-0.010, 0.005]	.468
	Nocturnal HR	-0.003 (0.012)	[-0.027, 0.022]	.833
	Stress	-0.003 (0.004)	[-0.010, 0.005]	.466
Positive Interactions	**Diary**			
	Restfulness of sleep	0.044 (0.043)	[-0.041, 0.130]	.309
	Stress	0.004 (0.002)	[0.001, 0.007]	.007**
	TST (hrs.)	-0.034 (0.030)	[-0.093, 0.025]	.257
	Stress	0.004 (0.002)	[0.000, 0.007]	.025*
	SOL (min.)	0.003 (0.004)	[-0.004, 0.010]	.342
	Stress	0.004 (0.002)	[0.001, 0.007]	.013*
	WUI	0.007 (0.230)	[-0.446, 0.461]	.975
	Stress	0.004 (0.002)	[0.001, 0.007]	.020*
	**Wearable**			
	TST (hrs.)	0.016 (0.036)	[-0.055, 0.087]	.656
	Stress	0.005 (0.002)	[0.001, 0.009]	.013*
	WUI	-0.051 (0.052)	[-0.155, 0.052]	.330
	Stress	0.005 (0.002)	[0.001, 0.008]	.014*
	Nocturnal HR	-0.008 (0.007)	[-0.021, 0.006]	.262
	Stress	0.005 (0.002)	[0.001, 0.008]	.015*

Note. TST = Total sleep time, SOL = Sleep onset latency, WUI = Wake-up index (no. awakenings / TST), HR = Heart rate, Social time = Amount of time spent with family/friends, Positive interactions = Number of daytime positive social interactions, B = Regression

coefficient for the fixed effect, SE = Standard error, CI = Confidence interval, * = $p < .05$, ** = $p < .01$, *** = $p < .001$, grand-mean-centered scores were used for self-reported restfulness of sleep and stress.
